# Foreign Substance Introduced into the Brain with Impunity

**Published:** 1881-08

**Authors:** 


### Foreign Substances Introduced into the Brain with Impunity.

At a recent meeting of the St. Louis Medical Society (*St. Louis Medical and Surgical Journal*, May, 1881, p. 566) a curious case was described of an insane convict who was in the habit of inserting wires, nails, etc., into his brain through an opening made in the skull by means of an awl. One of the wires was so long that it penetrated the brain-substance completely and struck against the skull on the other side. After the discharge of this convict from prison, he procured some morphia for the purpose of overcoming sleeplessness, and, taking an overdose, died.

A post-mortem examination was made by Dr. Carpenter, assisted by Dr. Sayer, of Leavenworth. In the substance of the brain the following foreign bodies were found: first, a wire four and three-fourths inches in length; second, a wire three and seven-eighths inches long; third, a wire six and three-fourths inches in length; a wire was removed from the middle lobe two and one-sixth inches long; one in the anterior lobe two and three-eighths inches long; a nail removed from the anterior lobe two and one-quarter inches in length; a needle removed from the middle lobe one and five-eighths inches in length. These were encysted in a manner in the substance of the brain, and apparently gave him no trouble whatever.

The patient had shown no psychical peculiarities during life which could be attributed to the presence of foreign bodies in the brain.

